# Changes to Neural Activation Patterns (c-fos Labeling) in Chinchilla Auditory Midbrain following Neonatal Exposure to an Enhanced Sound Environment

**DOI:** 10.1155/2018/7160362

**Published:** 2018-07-05

**Authors:** Lisa M. D'Alessandro, Robert V. Harrison

**Affiliations:** ^1^Department of Physiology, University of Toronto, Toronto, Canada M5S 1A8; ^2^Institute of Biomaterials & Biomedical Engineering, University of Toronto, Toronto, Canada M5S 3G9; ^3^The Auditory Science Laboratory, Program in Neurosciences and Mental Health, The Hospital for Sick Children, Toronto, Canada M5G 1X8; ^4^Department of Otolaryngology-Head and Neck Surgery, University of Toronto, Toronto, Canada M5G 2N2

## Abstract

Sensory brain regions show neuroplastic changes following deficits or experimental augmentation of peripheral input during a neonatal period. We have previously shown reorganization of cortical tonotopic maps after neonatal cochlear lesions or exposure to an enhanced acoustic environment. Such experiments probe the cortex and show reorganization, but it is unclear if such changes are intrinsically cortical or reflect projections from modified subcortical regions. Here, we ask whether an enhanced neonatal acoustic environment can induce midbrain (inferior colliculus (IC)) changes. Neonatal chinchillas were chronically exposed to a 70 dB SPL narrowband (2 ± 0.25 kHz) sound stimulus for 4 weeks. In line with previous studies, we hypothesized that such exposure would induce widening of the 2 kHz tonotopic map region in IC. To probe c-fos expression in IC (central nucleus), sound-exposed and nonexposed animals were stimulated with a 2 kHz stimulus for 90 minutes. In sound-exposed subjects, we find no change in the width of the 2 kHz tonotopic region; thus, our hypothesis is not supported. However, we observed a significant increase in the number of c-fos-labeled neurons over a broad region of best frequencies. These data suggest that neonatal sound exposure can modify midbrain regions and thus change the way neurons in IC respond to sound stimulation.

## 1. Introduction

The objective of this study is to examine the effects of a frequency-enhanced neonatal acoustic environment on c-fos expression patterns in the auditory midbrain (inferior colliculus). Many studies have revealed alterations in sensory cortical representation after deafferentation or enhanced sensory stimulation. Seminal work on such neuroplasticity has been made in visual cortex [1, 2], in somatosensory cortex [3], and in auditory cortex [4]. However, a question that arises is whether such plasticity is intrinsically cortical or whether it reflects, wholly or partially, reorganization at subcortical levels. In the auditory system of the cat, we have reported extensive reorganization of cortical tonotopic maps after neonatal cochlear hair cell lesions [5] and subsequently established that thalamic areas were similarly reorganized [6]. We have also observed tonotopic map changes in the inferior colliculus (IC) of the chinchilla auditory midbrain as a result of neonatal partial cochlear deafferentation [7]. In experiments where neonatal manipulations are made to the auditory system (either by sound deprivation or by augmentation), it is important to consider whether the experimental animal is altricious (born with an immature inner ear) or precocious (with hearing present at birth). In altricious rodent species, studies have revealed midbrain plasticity after augmented auditory stimulation during a neonatal period [8–14]. However, in precocious species such as the chinchilla (and humans), it remains unclear whether an enhanced acoustic environment in a neonatal period can induce neuroplastic change at the level of the auditory midbrain. We address this question in the present study. Newborn chinchilla pups are exposed to a 70 dB SPL narrowband (2 ± 0.25 kHz) sound stimulus for 4 weeks, and midbrain (IC; central nucleus) neural activity patterns are assessed using c-fos labeling techniques.

c-fos immunohistochemistry has been used to study neural activation patterns in a variety of brain regions following brief periods of enhanced sensory stimulation (e.g., [15–19]). In the auditory brain, c-fos labeling studies have been used in a range of species (cat [20], mouse [21], rat [22, 23], gerbil [24], and bat [25]), and we have previously reported on a successful c-fos protocol for the chinchilla [26]. We have shown that chronic stimulation with a tonal stimulus results in a band (sheet) of c-fos-labeled neurons in IC that corresponds to the tonotopic map functionally verified using single-unit electrophysiological recordings [26]. In the present study, we have used this c-fos expression method to reveal possible neuroplasticity in the IC of neonatal chinchillas exposed to a 70 dB SPL narrowband (2 ± 0.25 kHz) sound stimulus for 4 weeks. We compare the pattern and number of c-fos-labeled neurons in exposed subjects with age-matched control subjects. Based on previous studies that report neural overrepresentation of areas related to exposure stimuli, we hypothesize that our sound exposure will cause widening of the 2 kHz tonotopic map region in IC.

## 2. Materials and Methods

### 2.1. Overview of Experimental Protocol

**Figure 1:**
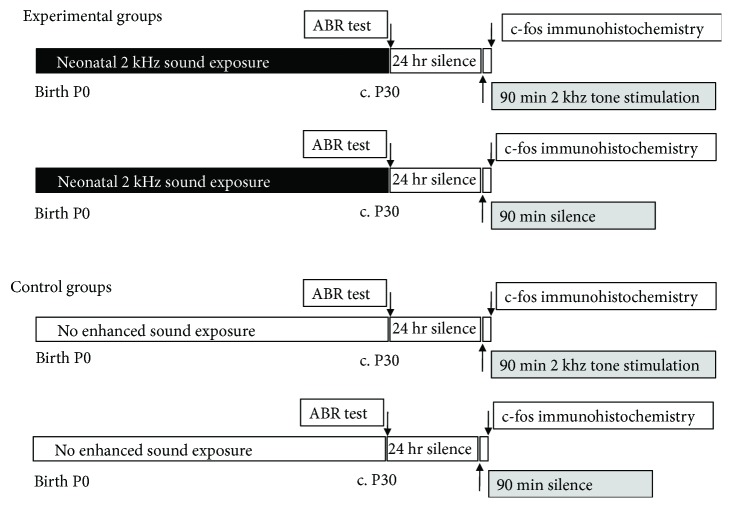
Experimental timelines for experimental neonatal sound-exposed groups and nonexposed controls.

**Figure 2:**
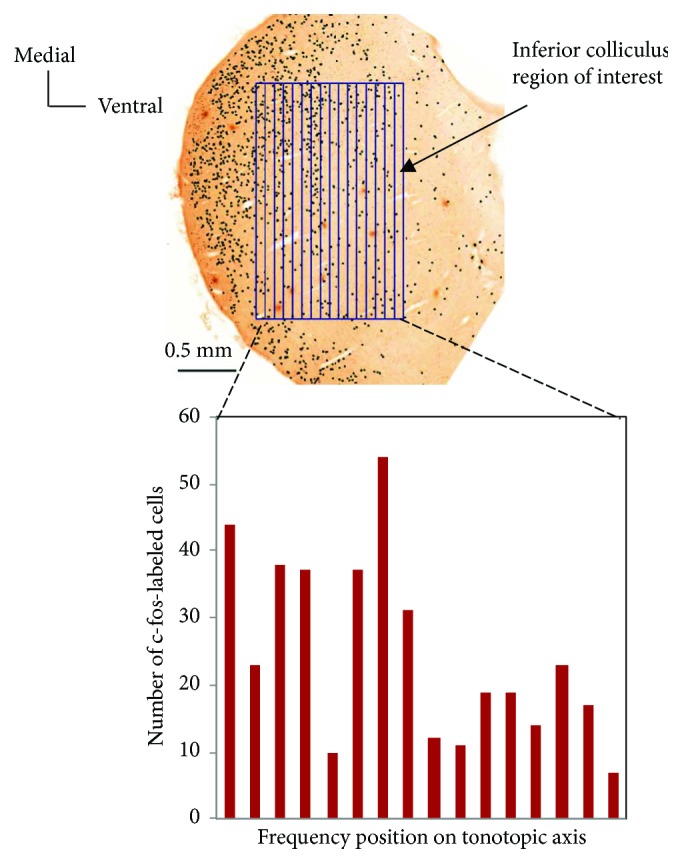
Quantification of c-fos immunolabeling results. The region of interest corresponds approximately to central nucleus of inferior colliculus. Scale bar 500 *μ*m. This is a representative sample from a subject stimulated for 90 min with a 2 kHz tone burst. The grid is superimposed to obtain cell counts shown in the lower bar graph. This upper histology image is reproduced from a methods description paper ([26]; fig. 4) with permission (CC BY-NC-ND license, under Crown Copyright).

Our aim is to examine the effects of a tone-enhanced acoustic environment on c-fos expression patterns in IC (central nucleus) of the chinchilla. The experimental protocols are summarized in Figures 1 and 2. For the main experimental group (upper panel of Figure 1), experimental subjects were placed in a 2 kHz frequency-augmented sound environment from birth (P0) for about 30 days (27–34-day range). At the end of this period, auditory brainstem responses (ABRs) to click stimuli were recorded to confirm normal hearing thresholds. This testing was carried out under anesthesia (ketamine 15 mg/kg, i.p.; xylazine 2.5 mg/kg, i.p.). In comparing ABR thresholds across frequency between sound-enriched and control animals, we have found and previously reported [27] no significant differences between enriched and control animals. Animals were kept overnight in a sound-attenuating booth. Subjects (unanesthetized) then either received a 90 min period of 2 kHz tone bursts (500 ms; 100 ms rise/fall) or remained in silence (second panel in Figure 1). There are two age-matched control groups with no neonatal sound augmentation (lower panels of Figure 1). The procedures for assessing neural activity with c-fos labeling were identical for all groups. Animals were anaesthetized (ketamine 15 mg/kg, i.p.; xylazine 2.5 mg/kg, i.p.) and perfused (transcardiac) for brain tissue fixation. IC specimens were processed to reveal c-fos protein expression in any activated neurons in IC. Cell count data were quantified as outlined in Figure 2.

### 2.2. Subjects

Twenty chinchillas (*Chinchilla laniger*; 10 females, 10 males) were obtained either on the day of birth (P0) or one day later (Roseneath Chinchilla, Ontario, Canada). Subjects were included in the study when auditory brainstem response (ABR) thresholds to broadband (47 *μ*s click) stimuli were <35 dB SPL. This initial screening was carried out in the anesthetized animal (ketamine 15 mg/kg, i.p.; xylazine 2.5 mg/kg, i.p.). Animals were randomly assigned to four groups (5 subjects in each) as outlined in Figure 1. There are two sound-exposed groups, where subjects were placed in an enhanced auditory environment for 27–34 days (average 30 days) and then either were stimulated with a 90 min period of 2 kHz tone bursts or received no further sound stimulation. There are two control groups, where subjects experienced no enhanced auditory environment and then had either a 90 min period of 2 kHz tone bursts (to induce frequency bands of fos-labeled cells) or no sound stimulation. Data from these two control groups were reported in a methods paper describing our c-fos labeling technique [26]. That previous publication has no scientific overlap with the present study. Some of the illustrative histological sections and control data have been reproduced or adapted in the present paper with permission (as stated in the figure legends). All procedures were approved under The Hospital for Sick Children Animal Care Committee protocols, following guidelines of the Canadian Council on Animal Care (CCAC).

### 2.3. Neonatal Sound Exposure

A pulsed (500 ms; 20 ms rise/fall; 1 s duty cycle) narrowband (2 ± 0.25 kHz) acoustic signal was presented continuously for c. 30 days in free field (Sony Micro Hi-Fi, CMT BX20i, coupled to Sony transducer Model #SS-CBX20, Miniato, Tokyo, Japan). The acoustic signal was calibrated to be 70 ± 5 dB SLP at ear level of the subject. Otherwise, the frequency spectrum of the ambient sound environment was flat, with no significant peaks. Animals did not exhibit abnormal behaviour when placed in this enhanced sound environment, and appeared to feed normally. We recorded no significant weight difference between control (mean ± SD; 169.9 ± 40.5 g) and neonatally sound-exposed groups (168.8 ± 35.1 g; *p* = 0.94, *t*-test).

### 2.4. c-fos Immunohistochemistry

#### 2.4.1. Acoustic Stimulation to Induce Tonotopic Frequency Bands of Labeled Cells in IC

Based on studies by others (in mouse: [21]; in rat: [17]) and our work in the chinchilla [26]), we used a 90-minute tonal stimulation duration to elicit tonotopic bands of c-fos-labeled neurons in IC. Subjects were exposed to a 2 kHz gated (100 ms rise/fall) signal for 90 minutes, calibrated to be 60 ± 5 dB SPL at the level of the ear.

#### 2.4.2. Histology Protocol

Following the end of the 90-minute period of stimulation (or silence for controls), subjects were perfused transcardially with saline, then with fixative (4% paraformaldehyde in 0.1 M phosphate buffer (PB) at 4°C). Whole brains were removed and kept overnight at 4°C in fixative. Coronal slices (40 *μ*m) were cut with a vibratome and treated to visualize the c-fos antigen. Sections were rinsed in PB (pH 7.4), incubated in 0.3% H_2_O_2_, rinsed in PB, incubated for 1 hr in blocking solution (0.1% bovine serum albumin; 0.2% Triton X-100; 2% goat serum in 0.1 M PB), and then further incubated for 48 hrs at 4°C in primary antibody (rabbit anti-Fos polyclonal IgG; diluted 1 : 500 in blocking solution). Sections were rinsed in PB, incubated for 1.5–2 hrs in biotinylated secondary antibody (goat anti-rabbit IgG; diluted 1 : 100 in blocking solution) at room temperature, then rinsed in PB, and reacted with ABC (Avidin-Biotin Complex in blocking solution, 1 : 50). Sections were incubated for 8 minutes in a 3,3′-diaminobenzidine (DAB) solution (0.05%); then, 0.001% H_2_O_2_ was added to reveal staining. Sections were rinsed with PB, mounted on gelatinized slides, dried, dehydrated through graded alcohol rinses, and cover-slipped.

#### 2.4.3. Image Analysis

**Figure 3:**
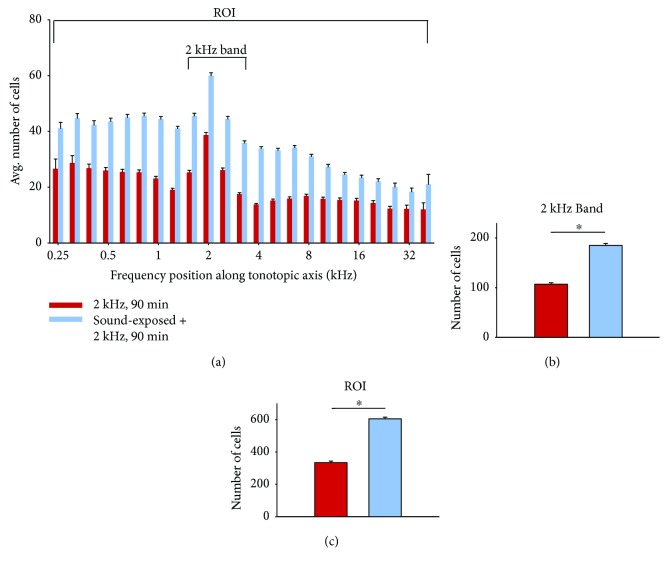
(a) Red bars (158 samples from 5 subjects) show average cell counts for subjects not reared with neonatal sound augmentation, but heard only the 2 kHz, 90 min probe. Average cell counts from subjects reared in the augmented acoustic environment, who then heard the 2 kHz, 90 min probe, are indicated in blue (194 samples from 5 subjects). One coronal slice produces 2 samples. Error bars show SEM. The difference in cell counts (b) within the 2 kHz band and (c) over the entire region of interest (ROI) are significant, ^∗^*p* < 0.001.

**Figure 4:**
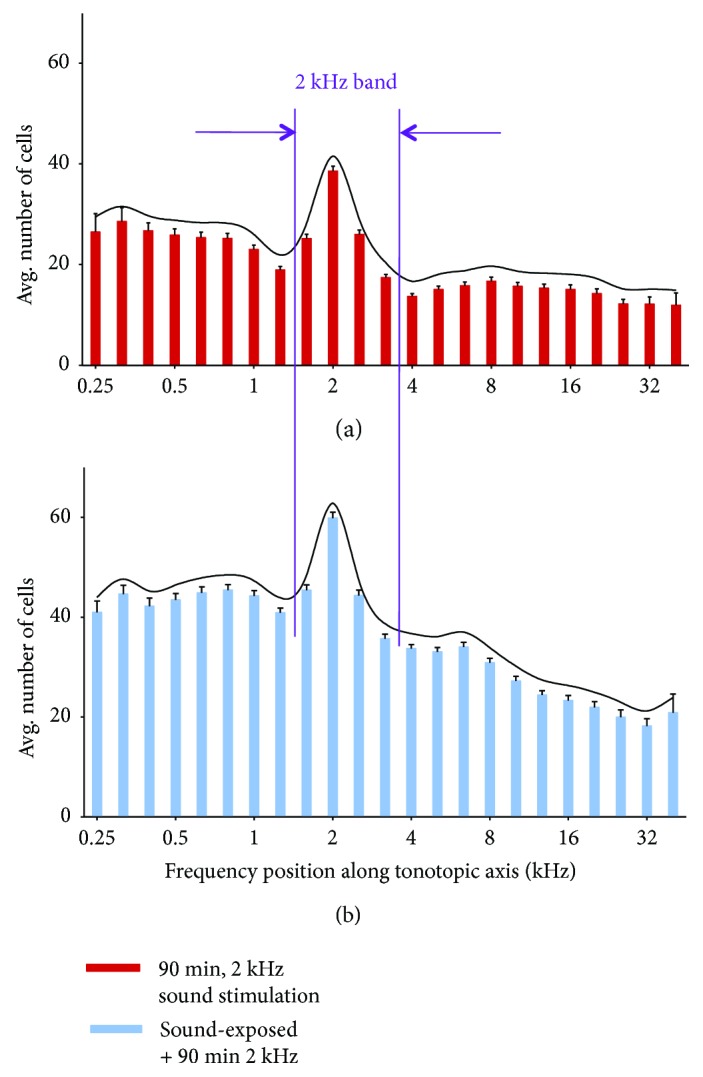
Comparison of the width of the 2 kHz tonotopic band in IC between subjects with neonatal 2 kHz sound exposure (b) and nonexposed controls (a). There is no significant difference in bandwidth between control (a) and sound-exposed (b) subjects. The upper graph has been adapted from a previously published methods description ([21]; fig. 3) with permission (CC BY-NC-ND license, under Crown Copyright).

**Figure 5:**
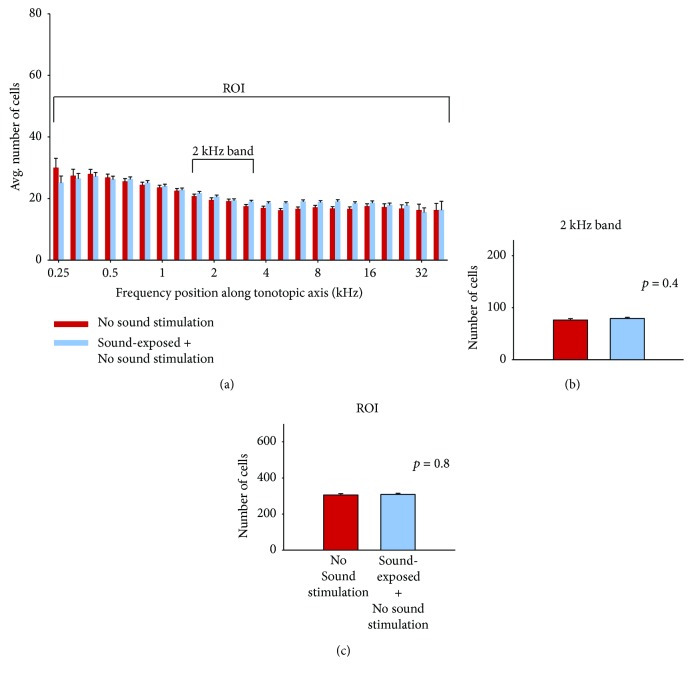
(a) Data in red show average counts of c-fos-labeled neurons for subjects who received neither the 4-week sound exposure from birth stimulus nor subsequent 2 kHz tone stimulation (199 samples from 5 subjects). Data for subjects having the neonatal exposure and then no additional sound stimulation are plotted in blue (249 samples from 5 subjects). Error bars show SEM. There is no significant difference in cell count in the estimated 2 kHz region (b) or over the entire region of interest (ROI; c). In (a), the “No Sound Stim” control data (red) has been reproduced from a previously published methods paper ([26]; fig. 3) with permission (CC BY-NC-ND license, under Crown Copyright).

Each midbrain IC was digitally imaged at 7x magnification using Mirax Scan™. Images were then analyzed using ImageJ™ as follows. Thresholds were selected that best captured the pattern of c-fos-labeled neurons across the entire colliculus. As illustrated in Figure 2, we defined a region of interest (ROI) that corresponds approximately to central nucleus of IC. By rotation of each specimen image, the axis of the labeled cell region was vertically positioned to align with the ROI grid. Thus, the ROI consisted of a series of contiguous, equal-sized rectangles that lay 200–400 *μ*m away from the dorsal and ventral edges of the colliculus and that are approximately parallel to the isofrequency tonotopic bands in IC. For each colliculus, we counted the number of labeled cells in each column of the ROI using a custom-made macro and present the data in histogram form. In Figures 3, 4, and 5, we scale the histogram abscissae to represent frequency position along the tonotopic axis of IC [26].

## 3. Results

**Figure 6:**
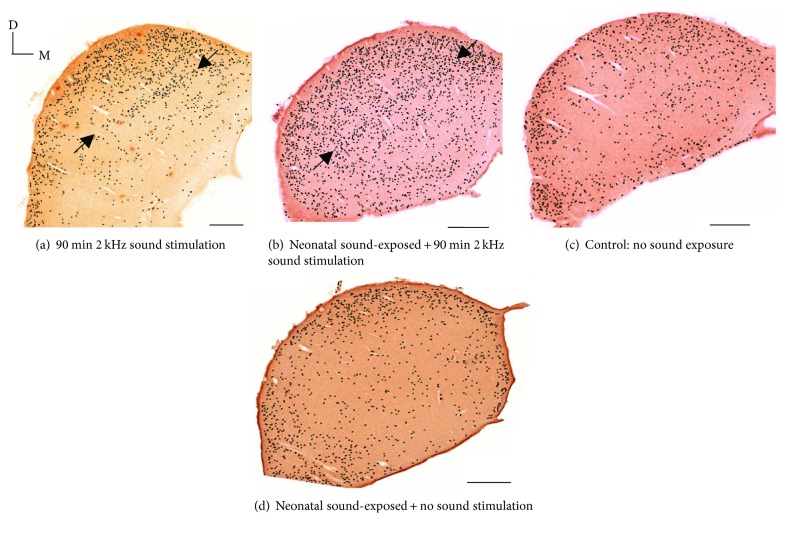
Representative patterning of c-fos-labeled cells in inferior colliculus by experimental group. Where present, arrows denote bands of labeled neurons. Scale bars indicate 500 *μ*m. The histology sections (a) and (c) are reproduced from a previously published methods paper ([26]; fig. 1) with permission (CC BY-NC-ND license, under Crown Copyright).

The qualitative examples in Figure 6 show typical patterns of c-fos labeling in the IC for the four experimental conditions.  Figure 6(a) shows c-fos labeling in IC following only 90 min of 2 kHz pure-tone sound stimulation, in a subject with no enhanced neonatal acoustic exposure. Note the labelling reveals a 2 kHz tonotopic band (between the arrows) as well as other background neural activation.  Figure 6(b) is from an animal reared for 4 weeks in the enhanced sound environment and is further stimulated with 2 kHz tone bursts for 90 min. Here, c-fos labeling shows the 2 kHz tonotopic band as well as an increase in c-fos-labelled cells over a broad range of the inferior colliculus. Compare this background activity with Figure 6(a) that had no enhanced neonatal exposure.  Figure 6(c) shows the typical low level of c-fos labelling in a subject with no sound exposure and no 90 min 2 kHz probe tone, and Figure 6(d) shows the IC of a subject reared for 4 weeks in the enhanced sound environmental and then had 90 minutes of silence before c-fos staining.

The effects of neonatal sound exposure on the activity patterns in IC (i.e., a comparison of the subject groups (a) and (b) of Figure 6) are shown in Figure 3. Here, quantification of cell counts from all subjects has been analyzed as described in Figure 2. There is a significant increase in both the numbers of c-fos-labeled cells both in the 2 kHz tonotopic band (3B) and over the entire region of interest (3C) compared with control subjects who were not reared in the enhanced sound enhancement (*p* < 0.001; data values are listed in Table 1).

Our original hypothesis was that the 2 kHz neonatal exposure would result in an enhanced midbrain neural representation of the 2 kHz tonotopic band. However, we report no difference in the width of the 2 kHz band. Our analysis of IC frequency bandwidth and experimental comparison is shown in Figure 4. The width of the 2 kHz tonotopic representation is measured as the distance between the two troughs, that is, regions of decreased neural labelling adjacent to the excited region.

The significant difference observed between the neonatal-exposed subjects and the nonexposed group (Figure 3; Table 1) is the overall increase in c-fos-labeled activity. Both groups have received the final 2 kHz “probe” stimulus before the activity is assessed. The neonatally sound-exposed subjects have in effect received 2 “treatments,” that is, the early sound exposure, and the 90 min period of 2 kHz tone bursts. To determine if the increase in c-fos-labeled neurons was due to sound exposure alone, we conducted a separate set of experiments in which subjects were exposed to the enhanced sound environment, then received no additional probe sound stimulation. Qualitative data are shown in Figures 6(c) and 6(d); note that there are no labelled tonotopic bands. Data quantification is illustrated in Figure 5. There is no difference in the number of labelled neurons either in the (estimated) 2 kHz band region or over the entire region of interest. These results suggest that there is no effect of sound exposure alone on basal levels of c-fos active cells but that the IC appears primed to be more generally active to subsequent acoustic stimulation.

## 4. Discussion

We have previously demonstrated a viable c-fos labelling protocol for the chinchilla [26]. We use this method here to study neuroplastic change in sound frequency representation in IC following rearing in an enhanced acoustic environment. Our focus on the midbrain is because so much evidence of developmental plasticity changes have reported on cortical reorganization, with little regard to possible subcortical plasticity.

Neonatal subjects reared for 4 weeks with a moderately intense 2 kHz sound exposure stimulus, then probed with a 90 min 2 kHz tone burst, and exhibited a significant increase in the number of c-fos-labeled neurons over the entire region of interest studied, including in the 2 kHz tonotopic band. We had hypothesized a widening of the 2 kHz tonotopic band, in line with other demonstrations of overrepresentation in neural areas correlated to exposure stimulus characteristics. However, we did not observe a change in the width of the 2 kHz band. This finding is important. At the cortical level in a precocious animal model, developmental plasticity results in considerable neural reorganization. At the auditory midbrain level, this window of developmental plasticity may well be closed.

However, we do observe subtle changes to stimulus activity levels in IC. In the subjects exposed to the neonatal 4-week enhanced sound stimulus and with no further probe sound stimulation, the c-fos expression patterns are no different from (age-matched) control animals having no experimental sound exposures. This is illustrated in Figure 5. These results suggest that the 4-week neonatal sound-exposure stimulus does not affect basal levels of neural activity as reflected in c-fos expression. Of interest, the data suggest a form of sensitization; that sound exposure alters how neurons in inferior colliculus respond to a subsequent somewhat prolonged (90 min) presentation of a pure-tone sound stimulus.

### 4.1. Use of c-fos Labeling Methodology

We sought a method to reveal global neural activation patterns in IC following experimental neonatal sound exposure. We selected c-fos immunohistochemistry from other immediate early genes for several reasons. First, basal expression levels of this protein are relatively low, allowing us to observe possible increases in neural activity following neonatal rearing in an enhanced sound environment. An alternative, the protein product of the NGFI-A immediate early gene, for example, is expressed at high basal activity levels [28]. Secondly, the c-fos protein is localized to the cell nucleus; thus, each instance of c-fos labeling indicates that that neuron has recently been activated. Various studies have used c-fos expression to examine how sensory stimulation can change neural activity patterns. In somatosensory cortex, vibrissae stimulation has been shown to increase c-fos expression in barrel cortex [16, 18]. In visual cortex, various studies have used c-fos labeling to explore light-induced neuroplastic change (e.g., [15, 19]). In the auditory system, midbrain changes to tonotopic maps have been demonstrated using c-fos methods (mouse: [21]; rat: [17]). A number of studies, including our own, report on the neural representation of sound frequency in IC following postnatal sound exposure using electrophysiological methods (e.g., [10–14, 26, 29]). However, the advantage of using c-fos immunolabeling is that it allows visualization of global neural activity patterns with cellular resolution.

### 4.2. IC Activity Patterns following Neonatal Sound Exposure

In regard to our neonatal sound exposure (2 kHz narrowband signal for 30 days at 70 dB SPL), one obvious question is whether it causes damage at the level of the cochlea. We suggest not because with a similar exposure, we have previously reported no change to ABR audiograms or to cochlear hair cell integrity as shown by scanning electron microscopy [27]. However, recent studies on noise exposure in rodent models have revealed inner hair cell synaptopathy and related spiral ganglion cell degeneration [30, 31]. These changes are not revealed by any monitoring of audiometric thresholds and have thus led to the notion of “hidden hearing loss.” Our own cochlear histology with scanning microscopy cannot detect synaptic pathology. So it is possible that the IC changes we observe may not be intrinsically midbrain but have some peripheral origin. An added point of interest is that Hickox and Liberman's group has linked noise-induced synaptopathy with tinnitus generation and hyperacusis [32]. A general sensitization of IC neural activity such as we demonstrate here is in-line, albeit tentatively, with a neural substrate for hyperacusis.

Subjects having the sound-exposure stimulus for 4 weeks and then no additional sound stimulation did not exhibit c-fos expression patterns that differed from age-matched controls (Figures 6(c) and 6(d); Figure 5). These results imply that sound exposure does not change basal levels of neural activity in inferior colliculus. Sound-exposed subjects who subsequently heard the 90 min 2 kHz tone burst exhibited increased c-fos expression levels, both near the 2 kHz region of interest and over a broad frequency range of inferior colliculus (Figures 6(b) and 3). Taken together, these data suggest that the sound-exposure stimulus alters the way in which neurons respond to a subsequent presentation of a sound stimulus, of sufficient duration, in the frequency range of the sound exposure stimulus (90 min 2 kHz tone burst).

Our main experimental finding is well captured in Figure 3. These data show that early sound-exposure stimulus alters the way in which neurons in IC respond to a subsequent presentation of a sound stimulus, and suggest a form of global sensitization. Our results resemble in some ways receptive field sensitization patterns in auditory cortex reported by Bakin and Weinberger and Weinberger [33, 34]. In their work on receptive field plasticity in auditory cortex, guinea pigs were subjected to various “sensitization training” stimulation paradigms, such that overall, subjects heard tone bursts at 80 dB for approximately 50 minutes. This sensitization training resulted in a broad, nonspecific increase in response across auditory cortical receptive fields; response changes were seen across a wide range of best frequencies up to 30 kHz [33]. Essentially, these sensitization results provide an example of pure-tone stimulation affecting change across a broad tonotopic frequency region and are an interesting comparison for the results presented herein.

The following question might be posed. Does the significant increase in the number of c-fos-labelled neurons, over a broad tonotopic frequency range in sound-exposed subjects, produce some change in hearing function? The answer has to be speculative, because we are far from understanding the exact relationship between neural activity patterns and perceived sounds. With that said, if we assume the pattern of excitation (across a broad tonotopic array) in IC is the direct substrate for higher-level sound processing, then the general increase in activity might translate into increase in loudness perception. This might perhaps contribute to some degree of hyperacusis. Secondly, if the general level of excitability is increased across all tonotopic areas, this might be considered “noise” in relation to a more specific coded sound signal. We could speculate that this would reduce the signal to noise in a neural representation of an acoustic signal. At an extreme, if this enhancement of IC neural activity is spontaneous and sustained (and perceived), it might contribute to some (broadband) tinnitus sensation.

In summary, the data presented here suggest that persistent exposure to an abnormal sound environment during an early postnatal period can alter neural activity patterns to sound in later life. Our data show such changes at the auditory midbrain level, but does not preclude the possibility of sensitization at brainstem or even cochlear levels of the system.

## Figures and Tables

**Table 1:** Average counts of c-fos-labelled neurons (±SEM) by the experimental group. *t*-tests were performed between pairs of groups, as indicated; the corresponding *p* values are reported.

Experimental group	Number of cells	
	2 kHz band	ROI
2 kHz, 90 min	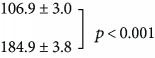	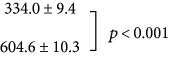
Sound-exposed + 2 kHz, 90 min		
No sound	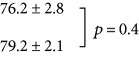	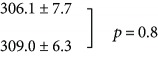
Sound-exposed + no sound		
